# Circulating Gut Microbe-Derived Metabolites Are Associated with Hepatocellular Carcinoma

**DOI:** 10.3390/biomedicines12091946

**Published:** 2024-08-26

**Authors:** Rakhee Banerjee, Chase J. Wehrle, Zeneng Wang, Jennifer D. Wilcox, Vinayak Uppin, Venkateshwari Varadharajan, Marko Mrdjen, Courtney Hershberger, Ofer Reizes, Jennifer S. Yu, Justin D. Lathia, Daniel M. Rotroff, Stanley L. Hazen, W. H. Wilson Tang, Federico Aucejo, J. Mark Brown

**Affiliations:** 1 Department of Cancer Biology, Lerner Research Institute, Cleveland Clinic, Cleveland, OH 44195, USA; banerjr2@ccf.org (R.B.); vxu14@case.edu (V.U.); varadhv@ccf.org (V.V.); mrdjenm2@ccf.org (M.M.); yuj2@ccf.org (J.S.Y.); 2 Center for Microbiome and Human Health, Lerner Research Institute, Cleveland Clinic, Cleveland, OH 44106, USA; wangz2@ccf.org (Z.W.); reizeso@ccf.org (O.R.); lathiaj@ccf.org (J.D.L.); hazens@ccf.org (S.L.H.); tangw@ccf.org (W.H.W.T.); 3 Department of Hepato-Pancreato-Biliary and Liver Transplant Surgery, Digestive Diseases and Surgery Institute, Cleveland Clinic, Cleveland, OH 44195, USA; wehrlec@ccf.org (C.J.W.); aucejof@ccf.org (F.A.); 4 Department of Cardiovascular and Metabolic Sciences, Lerner Research Institute, Cleveland Clinic, Cleveland, OH 44195, USA; kirsopj@ccf.org; 5 Department of Quantitative Health Sciences, Lerner Research Institute, Cleveland Clinic, Cleveland, OH 44195, USA; hershbc@ccf.org (C.H.); rotrofd@ccf.org (D.M.R.); 6 Center for Quantitative Metabolic Research, Cleveland Clinic, Cleveland, OH 44195, USA; 7 Case Comprehensive Cancer Center, Case Western Reserve University, Cleveland, OH 44195, USA; 8 Endocrinology and Metabolism Institute, Cleveland Clinic, Cleveland, OH 44195, USA; 9 Cleveland Clinic Foundation, Heart, Vascular and Thoracic Institute, Cleveland, OH 44195, USA

**Keywords:** nutrition, metabolism, microbiome, hepatocellular carcinoma, cirrhosis

## Abstract

Hepatocellular carcinoma (HCC) is the third leading cause of cancer death worldwide. The gut microbiome has been implicated in outcomes for HCC, and gut microbe-derived products may serve as potential non-invasive indices for early HCC detection. This study evaluated differences in plasma concentrations of gut microbiota-derived metabolites. Methods: Forty-one patients with HCC and 96 healthy controls were enrolled from surgical clinics at the Cleveland Clinic from 2016 to 2020. Gut microbiota-derived circulating metabolites detectable in plasma were compared between patients with HCC and healthy controls. Hierarchical clustering was performed for generating heatmaps based on circulating metabolite concentrations using ClustVis, with Euclidean and Ward settings and significant differences between metabolite concentrations were tested using a binary logistic regression model. Results: In patients with HCC, 25 (61%) had histologically confirmed cirrhosis. Trimethylamine (TMA)-related metabolites were found at higher concentrations in those with HCC, including choline (*p* < 0.001), betaine (*p* < 0.001), carnitine (*p* = 0.007), TMA (*p* < 0.001) and trimethylamine N-oxide (TMAO, *p* < 0.001). Notably, concentrations of P-cresol glucuronide (*p* < 0.001), indole-lactic acid (*p* = 0.038), 5-hydroxyindoleacetic acid (*p* < 0.0001) and 4-hydroxyphenyllactic acid (*p* < 0.001) were also increased in those with HCC compared to healthy controls. Hierarchical clustering of the metabolite panel separated patients based on the presence of HCC (*p* < 0.001), but was not able to distinguish between patients with HCC based on the presence of cirrhosis (*p* = 0.42). Conclusions: Gut microbiota-derived metabolites were differentially abundant in patients with HCC versus healthy controls. The observed perturbations of the TMAO pathway in HCC seem particularly promising as a target of future research and may have both diagnostic and therapeutic implications.

## 1. Introduction

Hepatocellular carcinoma (HCC) is the sixth most common cancer, the third leading cause of cancer-related mortality and the second most lethal cancer per case worldwide [1,2]. Both incidence and mortality of HCC continue to rise in Western countries, in large part due to the rising burden of both non-alcoholic and alcoholic steatohepatitis in the United States and Europe [3,4]. Cirrhosis and HCC are co-morbid in over 80% of cases, and thus any patient with cirrhosis or end-stage liver disease (ESLD) is at high risk for development of HCC [5]. Therefore, any study of HCC should account for the underlying architecture of the liver parenchyma.

HCC often originates in a background of chronic cirrhosis, which is initiated by viral hepatitis, alcohol abuse or metabolic dysfunction-associated steatohepatitis (MASH) [1,2,3]. Although HCC is one of the classic examples of a viral-driven cancer, there is also strong evidence that obesity is another major risk factor for developing HCC [1,2,3]. Today, the vast majority of HCC cases are indeed driven by either hepatitis B or C (HBV or HCV) infection [1]. However, with recent availability of highly effective therapies targeting the HBV and HCV viral pathogens, it is anticipated that viral-driven HCC will decline sharply over the next decade. Despite this exciting prediction with viral-associated HCC, the incidence of obesity-driven HCC is poised to rise at an alarming rate as the obesity epidemic continues to grow in developed countries. Although obesity is driven by many genetic and environmental determinants, alterations in the gut microbiome are central to the promotion of obesity.

The interplay between obesity, HCC, ESLD/cirrhosis and the gut microbiome is a field of emerging interest [4,5,6,7,8,9,10,11,12,13,14,15,16]. The gut–liver axis has, for example, been implicated in chronic injury to hepatocytes, leading to regenerative liver injury and HCC-inducing genetic damage [6]. Further studies have correlated alterations in the gut microbiome community with progression of HCC in those with background MASH-cirrhosis [7,8]. Indeed, alterations in gut-derived microbiota have been proposed as mechanisms to diagnose HCC in high-risk patients, and as potential markers for response to immunotherapy regimens [6,9,10]. Using sequencing-based methods to profile either the gut or HCC tumor microbiome, several groups have shown clear associations between microbial diversity and HCC initiation, progression and response to therapy [4,5,6,7,8,9,10,11,12,13,14,15,16]. However, despite clear links between the microbiome and liver cancer, the gut microbe-derived factors that could serve as either diagnostic or therapeutic targets are largely unknown. Our study provides key new information, given we are not relying on sequencing-based methodologies. Instead, we quantified the level of gut microbe-derived metabolic products in the circulation of HCC patients using stable isotope dilution liquid chromatography tandem mass spectrometry (LC-MS/MS).

It has long been assumed that for gut microbes to impact liver disease and HCC, bacterial cell wall products such as lipopolysaccharide (LPS) “leak” into the portal circulation to directly engage hepatic pattern-recognition receptors and drive chronic inflammation in the liver. Here, we investigate an alternative model, where gut microbial metabolism of nutrients common in high-fat diets results in the production of a wide variety of small molecule microbial metabolites that enter the systemic circulation [17,18,19]. While previous studies have demonstrated that variations in actual GI microbiota are associated with HCC, these have typically been identified either from oral or gut sequencing, and/or from multi-omics analyses of fecal/oral GI samples in patients with HCC [9,10,11,12,13,14,15,16]. Serologic analyses of metabolites known to be derived from the gut–liver axis are generally lacking in the literature. While current evidence is limited, this type of analysis may have the potential to identify potential targets for diagnosis and therapy. This study aimed to identify plasma gut microbe-associated metabolomic signatures that differ in the circulation of patients with HCC versus healthy patients, and compare patients who have HCC both with and without cirrhosis.

## 2. Materials and Methods

### 2.1. Study Population

Plasma samples from 41 patients with a diagnosis of HCC using OPTN classification were obtained from patients seen by a surgeon in the multi-disciplinary liver-tumor clinic from January 2016 to July 2020 (see Table 1). Only patients with OPTN 5 lesions and scheduled surgical resection were included in this cohort, and samples were obtained prior to surgery. This cohort was chosen to maximize our histologic understanding of the tumor, as well as to confirm or rule out any diagnosis of cirrhosis or other underlying liver disease. Patients were stratified by presence (n = 25) or absence (n = 16) of cirrhosis to obtain subgroup specific effect sizes for associations with metabolites. The etiology of cirrhosis included non-alcoholic steatohepatitis (9.8%), hepatitis B (9.8%), hepatitis C (34.1%), hemochromatosis (2.4%) and idiopathic (4,9%). Most patients were treatment naïve at the time of sample collection, though some had received loco-regional therapy as detailed below. Plasma samples from 96 healthy control patients were obtained from an institutional biorepository of patients known to have no history of malignancy, heavy alcohol use, hepatitis, cirrhosis or other known liver disease. These samples were collected at Cleveland Clinic, Cleveland, OH, USA. This study was approved by the Institutional Review Board IRB# 10-727 (CHAMPS) for healthy controls and 10-347 for HCC samples, and all patients signed informed consent prior to sample collection.

### 2.2. Liquid Chromatography Tandem Mass Spectrometry (LC-MS/MS)

For this study we wanted to broadly understand how gut microbe-derived metabolites originating from diverse dietary substrates are associated with HCC. Therefore, we used a stable-isotope-dilution liquid chromatography tandem mass spectrometry (LC-MS/MS) methods for the quantitative analysis of diverse bacterially derived metabolites that originate from dietary micronutrients such as choline and carnitine, as well as aromatic amino acids including phenylalanine, tyrosine and tryptophan. A total of 20 mL of human plasma was used for extraction of metabolites using the method described by Nemet and colleagues [19]. Briefly, 80 mL of isotope labeled internal standard mix was added to 20 uL of plasma, vortexed for 1 min, followed by spin down at 20,000 g, in 4 °C for 10 min. A total of 80 mL of supernatant was transferred to a MS vial with insert and 5 mL was used for LC-MS. The plasma concentration of gut microbe-associated metabolites originating from aromatic amino acids was recently described by Nemet and colleagues [19]. Trimethylamine (TMA) related metabolites were measured as previously descibed by Wang and colleagues [20]. Detailed substrate-producte maps of the microbe and host or co-metabolites in these diverse metaorganimsal nutrient metabolism pathways under invetigation here are also available in our previous publications [19,20,21].

### 2.3. Statistical Analysis

All values depicted are represented as mean concentrations with standard deviations. Significant differences between metabolite concentrations were tested using a binary logistic regression model with a Bonferroni adjustment for multiple hypothesis testing [21]. For all tests, an adjusted *p* < 0.05 was employed as the threshold for statistical significance. Hierarchical clustering was performed on the log fold change for each metabolite between the average concentrations of each group and that of healthy controls. Heatmaps were generated using Euclidean distance and Ward’s method using ClustVis software (https://biit.cs.ut.ee/clustvis/) [22].

## 3. Results

Detailed healthy control and HCC patient demographics are provided in Table 1. Healthy controls have an average age of 50.38 and average BMI is 28.06 for males and 27.46 for females. Of those with HCC, 25 (61.0%) had concurrent histology-confirmed cirrhosis, while the remaining 16 (39.0%) had histologically confirmed normal hepatic parenchymal architecture. The etiology of cirrhosis was Hepatitis C Virus (HCV, n = 14, 56%), Hepatitis B Virus (HBV, n = 4, 16%), non-alcoholic steatohepatitis (NASH, n = 4, 16%), idiopathic (n = 2, 8%) and hemochromatosis (n = 1, 4%). One-quarter (n = 11) of the entire HCC cohort had a history of diabetes mellitus, all of which was type 2 diabetes. An additional 96 patients with no known liver disease or cancer of any kind were included in the study as healthy controls. Limited background information (basic demographics) on these patients is available due to regulatory design of the study but they are independent, active persons who have not been diagnosed with any systemic medical conditions, including cirrhosis, any malignancy, diabetes, obesity or other potentially confounding diagnoses. Median follow-up for the HCC cohort was 1161 days, or 3.18 years. Seven patients (17.1%) received locoregional therapy prior to sample acquisition, including trans-arterial chemoembolization (TACE, n = 4), Yttrium-90 (Y90, n = 2) or both Y90 and TACE (n = 1). The rate of recurrence for all HCC patients was 36.5% (n = 15).

### 3.1. Gut Microbe-Derived Metabolites Are Altered in HCC

**Figure 1:**
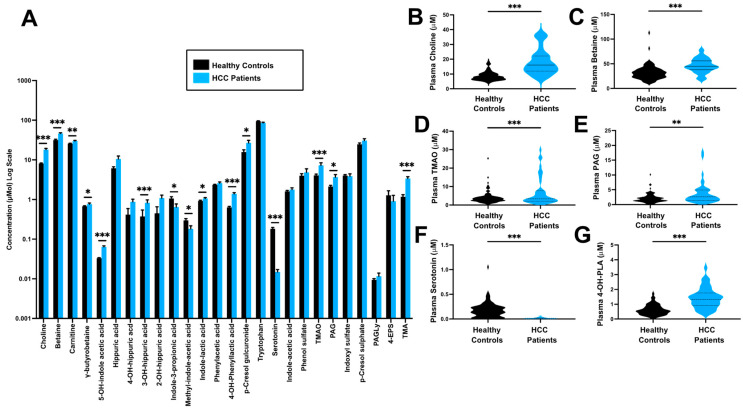
**Gut microbe-derived metabolites are altered in HCC.** Plasma levels of gut microbe-associated metabolites were quantified using stable isotope dilution tandem mass spectrometry (LC-MS/MS) in patients with HCC (n = 41) or healthy controls (n = 96). (**A**) Relative mean plasma concentrations of all metabolites measured. (**B**–**G**) Violin plots for individual metabolites that were most strikingly altered in HCC patients compared to controls. Significant differences between groups were tested using a binary logistic regression model with a Bonferroni adjustment for multiple hypothesis testing. Abbreviations: PAG, phenylacetylglutamine; PAGly phenylacetylglycine; PCS, p-cresol sulfate; TMA, trimethylamine; TMAO, trimethylamine N-oxide; 4-EPS, 4-ethylphenyl sulfate; 4-OH-PLA, 4-hydroxyphenyllactic acid. Stars in this figure indicate the level of statistical significance, * indicates a *p*-value less than 0.05, ** indicates a *p*-value less than 0.01, and ***, indicates a *p*-value less than 0.001.

To understand whether the circulating levels of gut microbe-associated metabolites are altered in HCC, we applied a targeted stable isotope dilution liquid chromatography tandem mass spectrometry (LC-MS/MS) method to plasma collected either from healthy controls that had no documented history of any malignancy (n = 96) or patients with HCC (n = 41). When comparing HCC patients to healthy controls, 14/25 (56%) of the metabolites investigated were significantly different (Figure 1 and Table 2). Notably, 10/15 were increased in patients with HCC when compared to healthy controls (Adjusted, *p* < 0.05). Metabolites that were increased in HCC patients included choline (18.1 vs 8.5 µmol/L, *p* < 0.001), betaine (46.0 vs 31.7 µmol/L, *p* < 0.001), *L*-carnitine (29.5 vs 25.6 µmol/L, *p* = 0.007), g-butyrobetaine (0.075 vs 0.67 µmol/L, *p* = 0.029), 5-hydroxyindole acetic acid (0.064 vs 0.033 µmol/L, *p* < 0.001), methyl-indole-acetic acid (0.18 vs 0.29 µmol/L, *p* = 0.030), indole-lactic acid (1.06 vs 0.91 µmol/L, *p* = 0.038), 4-hydroxyphenyllactic acid (4-OH-PLA,1.41 vs 0.63 µmol/L, *p* < 0.001), P-cresol glucuronide (26.9 vs 15.8 µmol/L, *p* = 0.032), trimethylamine-N-oxide (TMAO, 7.11 vs 3.99 µmol/L, *p* < 0.001), phenylacetylglutamine (PAG, 3.66 vs 2.11 µmol/L, *p* = 0.002) and trimethylamine (TMA, 3.30 vs 1.17 µmol/L, *p* < 0.001). As shown in Figure 1B–G, several of the metabolites were elevated in HCC patients (choline, betaine, g-butyrobetaine, *L*-carnitine, TMA and TMAO) and these metabolites are in a common metaorganismal metabolic pathway, whereby gut microbes can initially metabolize dietary substrates such as choline, g-butyrobetaine and *L*-carnitine into the primary gut microbe-derived metabolite TMA. Conversely, two molecules were reduced in HCC patients compared to controls, including indole-3-propionic acid (0.63 vs 1.03 µmol/L, *p* = 0.038) and serotonin (0.015 vs 0.180 µmol/L, *p* < 0.001). The difference in metabolite abundance between HCC and healthy controls is shown in Figure 1 and Table 2.

### 3.2. Impact of Background Liver Morphology on Circulating Gut Microbe-Derived Metabolites

**Figure 2:**
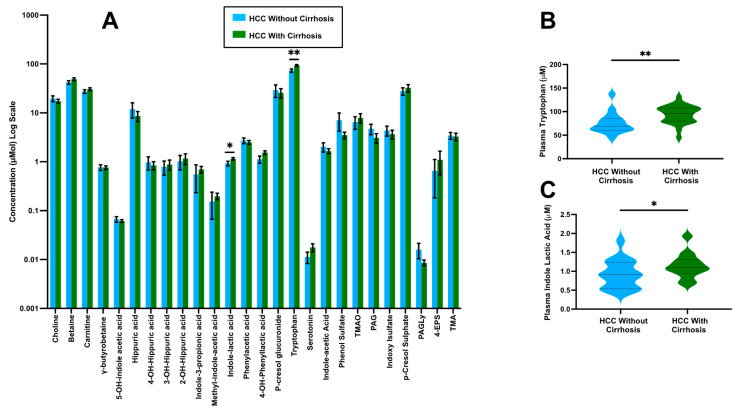
**Gut microbe-derived metabolites in HCC patients with or without underlying cirrhosis.** Plasma levels of gut microbe-associated metabolites were quantified using stable isotope dilution tandem mass spectrometry (LC-MS/MS) in patients with HCC with underlying cirrhosis (n = 25) and HCC patients without cirrhosis (n = 16). (**A**) Relative mean plasma concentrations of all metabolites measured. (**B**,**C**) Violin plots for individual metabolites that were significantly altered in HCC patients with or without underlying cirrhosis. Significant differences between groups were tested using a binary logistic regression model with a Bonferroni adjustment for multiple hypothesis testing. Abbreviations: PAG, phenylacetylglutamine; PAGly, phenylacetylglycine; TMAO, trimethylamine N-oxide; 4-EPS, 4-ethylphenyl sulfate. Stars in this figure indicate the level of statistical significance, * indicates a *p*-value less than 0.05, ** indicates a *p*-value less than 0.01.

We next investigated the hypothesis that alterations in gut-derived metabolites were arising from HCC-associated changes in liver function due to background cirrhosis. Here, the concentration of gut microbe-derived metabolites was compared within the HCC cohort based upon the presence of cirrhosis versus healthy background liver (Figure 2 and Table 3). We observed significant differences in the mean concentrations of only two metabolites—tryptophan (93.9 vs. 75.5 µmol/L, *p* = 0.009) and indole-lactic acid (1.15 vs 0.92 µmol/L, *p* = 0.033), which were both found to be elevated in patients with both HCC and cirrhosis compared with patients who have HCC and normal liver parenchyma (Figure 1 and Table 3).These two metabolites play an important role in immune tolerance and response to anticancer drugs; thus, these data indicate that background cirrhosis does not have a strong impact on the circulating levels of the gut microbe-derived metabolites investigated.

### 3.3. Hierarchical Clustering Clearly Distinguishes Healthy Controls from HCC Patients

**Figure 3:**
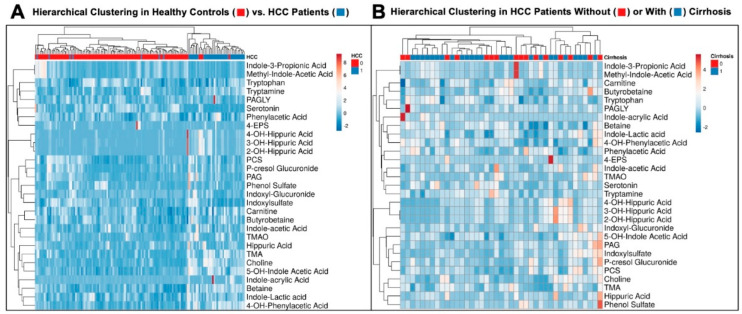
**Hierarchical clustering clearly distinguishes healthy controls from HCC patients.** Plasma levels of gut microbe-associated metabolites were quantified using stable isotope dilution tandem mass spectrometry (LC-MS/MS) in patients with HCC and healthy controls. Shown here are Heatmaps demonstrating differential metabolite abundance. (**A**) Gut microbiota-derived metabolites can distinctly classify patients without HCC (healthy controls, red) from patients with HCC (blue). (**B**) Gut microbiota -derived metabolites are not differentially expressed in patients with HCC based on the quality of their underlying liver parenchyma.

Next, hierarchical clustering was performed to assess whether the overall metabolite profile was different between those with HCC and healthy controls, and a clear distinction was observed (*p* < 0.001—Figure 3A). An additional analysis was performed to determine whether patients with HCC and cirrhosis clustered in a manner that distinguished them from those with HCC without cirrhosis, but no significant differences were observed (Figure 3B). These results indicate that levels of the metabolites concentrations measured here were not dramatically impacted by underlying cirrhosis.

## 4. Discussion

The gut–liver access is increasingly implicated in the regulation of chronic liver disease, yet its relevance to primary liver cancers is only beginning to be explored. This work demonstrates that circulating levels of several gut microbe-derived metabolites (TMA, TMAO, indole-3-propionic acid, 5-hydroxyindole acetic acid, methyl indole acetic acid, indole lactic acid, 4-hydroxyphenyllactic acid, phenylacetylglutamine) are altered in HCC patients compared to healthy controls. Although it is well known that reorganization of the gut microbiome assessed by fecal 16S rRNA or shotgun metagenomic sequencing is associated with diverse liver diseases such as MASLD, MASH, ALD and HCC [23,24,25,26,27,28,29,30,31], there is very little information on gut microbe-associated metabolomic signatures of these same liver diseases. Here we show that quantifying circulating metabolites originating from gut microbe-driven metabolism can distinguish patients with HCC from healthy controls. However, using this approach, this same set of metabolites was not able to separate patients with HCC by the presence of underlying cirrhosis. This study provides important proof of concept that gut microbe-focused metabolomic approaches may hold promise for improved detection of HCC and could provide clues into mechanistic links between metaorganismal nutrient metabolism and the development of HCC.

The liver represents the primary site of metabolism for almost all nutrients and xenobiotics we ingest. Gut microbe-derived metabolites are produced initially in the gut, absorbed into portal circulation and then delivered to the liver for downstream metabolism by the host [17,18]. As such, altered concentrations of gut microbe-derived metabolites in the peripheral circulation are a result of combinations of compounds that originate from both microbe- and host-driven metabolic processes. It is reasonable to assume that combined reorganization of the gut microbiome community coupled together with dysfunctional hepatic metabolism may together be reflected in the plasma concentrations of metabolites linked to microbial metabolism. This study represents one of the first studies to perform a targeted metabolomic investigation into metabolites that originate from the gut microbial endocrine organ [17,18]. Using this approach, we show that alterations in gut microbial metabolism of choline/L-carnitine, tyrosine, phenylalanine and tryptophan are associated with HCC. These findings warrant further investigation into whether altered gut microbial metabolism is simple associated with, or instead can be causally linked to HCC. Although there are very few examples of gut microbe-derived metabolites that have been causally linked to human disease, one powerful example can be highlighted by the metaorganismal TMAO pathway found to be altered in HCC patients here.

The TMAO pathway is initiated when nutrient precursors commonly found in high-fat foods (phosphatidylcholine, choline, *L*-carnitine and g-butyrobetaine) are metabolized by substrate-selective bacterial enzyme systems including *cutC/D* (choline-specific), *yeaW/X*, *bbu* and *gbuA-E* (g-butyrobetaine specific), *cntA/B* (L-carnitine specific) and likely others [32,33,34,35,36]. Once TMA is generated by bacteria, it can be rapidly transported via the portal vein to the liver where it can be subsequently metabolized by the host liver enzyme monooxygenases yielding TMAO [37]. The TMAO pathway has been shown to alter cholesterol and bile acid metabolism and alter cholesterol transporters in the liver and intestine that have been previously linked to liver cancer [38,39,40]. TMAO-mediated processes have also been specifically shown to regulate liver–microbiome crosstalk and thus promote or reduce liver fibrosis in MASH [41]. Most interestingly, a 2018 study by Liu et al. demonstrated a higher concentration of TMAO in patients with primary liver cancers, findings which are further supported by this work [42]. Although our studies here have focused on HCC associations, TMAO and related metabolites have also been studied in other liver disease etiologies. In agreement with our finding here, several studies have shown that plasma TMAO levels are elevated in subjects with metabolic dysfunction-associated fatty liver disease (formerly called non-alcoholic fatty liver disease) [42,43,44,45]. In contrast, we recently reported that in subjects with alcoholic hepatitis, plasma TMAO is reduced with reciprocal elevations in TMA [46]. This reciprocal alteration in TMA and TMAO in alcohol-associated hepatitis is due at least in part to reduced expression of the host hepatic enzyme flavin-containing monooxygenase 3 (FMO3) that is responsible for converting TMA to TMAO [46]. Collectively, there is strong emerging evidence that the metaorganismal TMAO pathway is altered in liver disease, but the mechanisms underlying this as well as therapeutic potential for TMAO-lowering drugs require further investigation.

Gut microbe-associated metabolites that were at higher concentrations in the HCC cohort compared to healthy controls were not differentially abundant within the HCC cohort stratified by the presence of underlying cirrhosis. Metabolites in the TMAO pathways have been linked to inflammatory or fibrotic regulation in cirrhotic pathways [36,37,38]. However, nearly identical levels of TMAO pathway metabolites were found in the cirrhotic and non-cirrhotic HCC patients here. Importantly, the metabolic signatures classified patients by presence of malignancy despite the heterogenous presence of cirrhosis in this population, which may point to a diagnostic use for this difference, especially in the high-risk cirrhotic cohort. Further, given the findings of Zhou et al. that direct TMAO infusion in the portal system can reduce liver fibrosis [41], mechanistic studies examining the impact of TMAO pathway metabolites on HCC outcomes may be of interest in ascertaining the utility of this axis moving forward.

In addition to clear alterations in the TMAO pathway, this study also found elevated levels of other interesting metabolites that originate from gut microbes in the HCC group. This includes p-cresol glucuronide, a conjugated form of p-cresol, a molecule with known uremic toxicity that results from bacterial metabolism of tyrosine [47,48]. P-cresol glucuronide also plays an anti-inflammatory role mediated by TLR4 [49], a key signaling pathway known to modulate immune response in both metabolic and alcoholic liver disease [50,51,52]. However, while p-cresol glucuronide was elevated in the HCC cohort, no differences were found within the HCC cohort by presence of cirrhosis, perhaps implicating the TLR4 pathway in the carcinogenic process. Interestingly, however, p-cresol glucuronide’s close relative, p-cresol sulfate was not elevated in HCC patients. The preferentially increased level of the glucuronidated derivative may warrant further investigation, as one might expect p-cresol sulfate to covary with this closely related pathway.

As with any study, there were limitations that warrant consideration. Most notably this study investigated a single time point of collection of the plasma. All patients had active malignancy at the time of collection, but the longitudinal impact of various treatments, including curative-intent surgical resection, cannot be ascertained. Since we did not also have samples post-resection, the concentration change after a theoretically curative surgery is likely to change, but we cannot make definitive comment. The healthy control population is known to be collected from patients without any cancer diagnosis, but additional information is not available due to nature of the samples and regulatory restrictions. Another clear limitation here is the relatively small sample size of this cohort. Validation of our findings in larger cohorts with sufficient numbers of underlying metabolic or viral associated tumors is an obvious next step. Another important limitation of this study is that the HCC patients were compared to healthy controls that did not have underlying liver disease. Follow up studies will include important liver disease controls without HCC including those with MASH, alcoholic hepatitis, as well as viral hepatitis with or without cirrhosis. Sequencing of stool was not available for these patients, which means that we could not correlate the circulating concentrations with differences in the actual bacterial colonization. However, this limitation is not particularly concerning given the fact that 16S and shotgun metagenomic sequencing very poorly predict circulating levels of gut microbe-derived metabolites [53,54]. Even with these noted limitations, we expect these results to inform downstream gut microbiome-focused metabolomic investigation in HCC and other cancers.

## 5. Conclusions

Gut microbiota-derived metabolites are differentially abundant in the peripheral plasma of patients with HCC compared to healthy controls, including striking changes in metabolites of the TMAO pathway. This difference was not observed between those with HCC with and without cirrhosis. Overall, these results may present an opportunity for improved diagnostic utility and could inform future interventional studies. The results from this study may also inform downstream investigation into how gut microbial metabolism can impact HCC initiation, progression and/or response to therapy. There is a growing appreciation that alterations in the gut microbiome may underlie liver disease progression towards HCC, and our study provides the first glimpse into the gut microbiota-derived metabolomic signatures of HCC. Although the current study only allows us to associate gut microbial metabolites with HCC risk, these finding could provide new clues into new therapeutic targets in HCC. For example, there are several emerging examples of small molecule inhibitors targeting gut microbial enzymes to lower the level of disease-associated metabolites [55,56]. In fact, we have recently developed mechanism-based small molecule inhibitors for the bacterial choline trimethylamine lyase *cutC/D* [57], and have shown that these bacterially targeted drugs can very effectively protect against common diseases associated with non-viral HCC including obesity [58], alcohol-associated [46] and metabolic dysfunction-associated fatty liver disease (MAFLD) in mice [58]. As a logical next step, it will be important to test the efficacy of these bacterially targeted TMAO-lowering drugs in preclinical animal models of HCC. Additional work is now required to determine whether the HCC-associated metabolites identified here are simply biomarkers of disease or potentially causally related.

## Figures and Tables

**Table 1:** (**a**) Patient demographics for healthy control subjects. (**b**) Patient demographics for subjects with hepatocellular carcinoma (HCC).

(**a**)			
		**Total (n = 96)**	
**Average Age**		50.38	
**Male Sex**		44	
**Race**			
White		32 (72.72%)	
Black		11 (25.0%)	
Asian		1 (2.27%)	
**Average BMI** (kg/m^2^)		28.06	
**Female Sex**		50	
**Race**			
White		45 (90.0%)	
Black		5 (10.0%)	
**Average BMI** (kg/m^2^)		27.46	
(**b**)			
	**Total HCC (n = 41)**	**HCC + Cirrhosis (n = 25)**	**HCC Alone (n = 16)**
**Median Age** (IQR)	68 (62.5–76.5)	66 (62–72)	73 (62.5–79.3)
**Male Sex**	13	7 (28%)	6 (37.5%)
**Race**			
White	34 (82.9%)	19 (76%)	15 (93.7%)
Black	4 (9.8%)	4 (16%)	-
Asian	3 (7.3%)	2 (8%)	1 (6.3%)
**BMI** (kg/m^2^)	27.3 (24–30.7)	27.3 (24–31.3)	27.6 (25.7–30.7)
**Etiology of Cirrhosis**			-
NASH	4 (9.8%)	4 (16%)	
Hepatitis B	4 (9.8%)	4 (16%)	
Hepatitis C	14 (34.1%)	14 (56%)	
Idiopathic	2 (4.9%)	2 (8%)	
Hemochromatosis	1 (2.4%)	1 (4%)	
**Diabetes**	11 (26.8%)	8 (32%)	3 (18.8%)
**No. Lesions** Median (IQR)	1 (1–2)	1 (1–2)	1 (1–3)
**Size Largest Lesion** Median cm (IQR)	4 (2.5–6)	3 (2–4)	10 (4.3–14.3)
**Treatment Pre-Sample Collection**			
Y90	3 (7.3%)	2 (8%)	1 (6.3%)
TACE	4 (9.8%)	2 (8%)	2 (12.6%)
None	34 (82.9%)	21 (84%)	13 (81.1%)
**Grade of Differentiation**			
Well	4 (9.8%)	-	4 (25%)
Moderate	34 (82.9%)	24 (96%)	10 (62.5%)
Poor	3 (7.3%)	1 (4%)	2 (12.6%)
**Vascular Invasion**	30 (73.2%)	20 (80%)	10 (62.5%)
**Recurrence**	15 (36.6%)	9 (36%)	6 (37.5%)

Age, race and BMI are expressed as count in percent; gut microbe-associated metabolites are altered in HCC patients compared to healthy controls. All the variables are expressed as count in percent. Age, number and size of lesions is presented as median (IQR).

**Table 2:** **Select gut microbe-derived metabolites are altered in HCC patients compared to healthy controls.** Circulating concentration of gut-derived metabolites in patients with hepatocellular carcinoma split by background liver morphology. Between-group means compared using binary logistic regression model with the Tukey-b adjustment for multiple testing.

	HCC (n = 41)	Healthy Control (n = 96)	*p*-Value
**Choline**	18.10 (8.54)	8.49 (2.75)	**<0.001**
**Betaine**	46.0 (14.5)	31.70 (14.1)	**<0.001**
**Carnitine**	29.5 (9.2)	25.60 (6.2)	**0.007**
**g-butyrobetaine**	0.75 (0.33)	0.67 (0.22)	**0.029**
**5-OH-indole acetic acid**	0.064 (0.025)	0.033 (0.010)	**<0.001**
**Hippuric acid**	9.61 (12.2)	6.27 (5.65)	0.124
**4-OH-hippuric acid**	0.89 (0.037)	0.40 (1.68)	0.106
**3-OH-hippuric acid**	0.84 (0.98)	0.36 (1.48)	**<0.001**
**2-OH-hippuric acid**	1.09 (1.32)	0.43 (1.91)	0.108
**Indole-3-propionic acid**	0.63 (0.83)	1.03 (1.08)	**0.028**
**Methyl-indole-acetic acid**	0.18 (0.23)	0.29 (0.30)	**0.030**
**Indole-lactic acid**	1.06 (0.36)	0.91 (0.42)	**0.038**
**Phenylacetic acid**	2.59 (1.19)	2.36 (0.68)	0.105
**4-OH-Phenyllactic acid**	1.41 (0.70)	0.625 (0.36)	**<0.001**
**p-Cresol glucuronide**	26.9 (28.1)	15.8 (20.3)	**0.032**
**Tryptophan**	86.7 (22.3)	94.9 (20.7)	0.056
**Serotonin**	0.015 (0.014)	0.18 (0.15)	**<0.001**
**Indole-acetic acid**	1.79 (1.22)	1.58 (0.81)	0.177
**Phenol Sulfate**	4.69 (7.09)	3.94 (4.66)	0.508
**Trimethylamine N-oxide (TMAO)**	7.11 (7.72)	3.99 (3.26)	**<0.001**
**Phenylacetylglutamine (PAG)**	3.66 (3.73)	2.11 (1.50)	**0.002**
**Indoxyl sulfate**	3.80 (3.65)	4.03 (2.20)	0.748
**p-Cresol sulfate**	30.7 (23.9)	24.70 (19.0)	0.224
**Phenylacetylglycine (PAGLY)**	0.011 (0.014)	0.0096 (0.0064)	0.281
**4-ethylphenyl sulfate (4-EPS)**	0.88 (2.33)	1.25 (3.78)	0.487
**Trimethylamine (TMA)**	3.30 (2.42)	1.17 (1.38)	**<0.001**

**Table 3:** **Cirrhosis has minimal impact on circulating levels of gut microbe-associated metabolites in HCC.** Circulating concentration of gut-derived metabolites in patients with hepatocellular carcinoma split by background liver morphology of HCC alone versus HCC in the background of underlying cirrhosis.

	Cirrhosis + HCC (n = 25)	HCC Alone (n = 16)	*p*-Value
**Choline**	17.3 (7.67)	19.3 (9.90)	0.321
**Betaine**	48.7 (14.7)	41.9 (13.7)	0.078
**Carnitine**	30.6 (9.54)	27.6 (8.53)	0.300
**g-butyrobetaine**	0.75 (0.30)	0.75 (0.39)	0.776
**5-OH-indole acetic acid**	0.061 (0.019)	0.067 (0.032)	0.618
**Hippuric acid**	8.46 (9.70)	11.4 (15.5)	0.162
**4-OH-hippuric acid**	0.84 (0.76)	0.96 (1.09)	0.744
**3-OH-hippuric acid**	0.88 (1.00)	0.74 (0.92)	0.539
**2-OH-hippuric acid**	0.866 (1.00)	0.78 (0.94)	0.804
**Indole-3-propionic acid**	0.699 (0.51)	0.15 (0.32)	0.645
**Methyl-indole-acetic acid**	0.32 (0.14)	1.00 (1.24)	0.620
**Indole-lactic acid**	1.15 (0.33)	0.92 (0.38)	**0.033**
**Phenylacetic acid**	2.50 (1.07)	2.73 (1.37)	0.546
**4-OH-Phenyllactic acid**	1.54 (0.62)	1.21 (0.79)	0.079
**p-Cresol glucuronide**	28.6 (26.5)	28.5 (31.2)	0.507
**Tryptophan**	93.9 (19.4)	75.5 (22.4)	**0.009**
**Serotonin**	0.17 (0.16)	0.011 (0.011)	0.131
**Indole-acetic acid**	1.67 (0.90)	2.00 (1.61)	0.414
**Phenol Sulfate**	3.40 (2.61)	6.71 (10.78)	0.141
**Trimethylamine N-oxide (TMAO)**	7.7 (8.27)	6.17 (6.93)	0.412
**Phenylacetylglutamine (PAG)**	3.06 (3.31)	4.61 (6.93)	0.183
**Indoxyl sulfate**	3.56 (3.63)	4.61 (4.26)	0.652
**p-Cresol sulfate**	32.4 (27.2)	28.1 (17.8)	0.787
**Phenylacetylglycine (PAGLY)**	0.0085 (0.005)	0.15 (0.21)	0.136
**4-ethylphenyl sulfate (4-EPS)**	0.97 (2.45)	2.29 (6.03)	0.372
**Trimethylamine (TMA)**	1.05 (2.66)	0.611 (1.74)	0.632

## Data Availability

The original contributions presented in the study are included in the article, further inquiries can be directed to the corresponding author.
